# Early life stress-induced depression reveals distinct region-specific modulation of unfolded protein response genes in the prefrontal cortex and hippocampus of rats

**DOI:** 10.3389/fpsyt.2026.1747106

**Published:** 2026-03-05

**Authors:** Andrew Kirk Griffin, Bhaskar Roy, Yogesh Dwivedi

**Affiliations:** Department of Psychiatry and Behavioral Neurobiology, University of Alabama at Birmingham, Birmingham, AL, United States

**Keywords:** early-life stress, environmental enrichment, hippocampus, major depressive disorder, prefrontal cortex, unfolded protein response

## Abstract

**Introduction:**

Early-life stress (ELS) has been implicated in the onset of major depressive disorder (MDD) in adulthood. Recent studies suggest that the unfolded protein response (UPR), a cellular response to stress, could be a precipitating factor in depressive symptoms.

**Methods:**

The present study examined the expression of genes associated with UPR in the prefrontal cortex (PFC) and the hippocampus using a rodent model of ELS-induced depression, in which male pups experienced maternal separation (MS) with or without subsequent environmental enrichment (MS-E). Expression of UPR genes was determined by quantitative polymerase chain reaction using rat-specific primers.

**Results:**

Of the six key UPR genes (*Xbp1, sXbp1, Atf4, Atf6, Grp94*, and *Chop*) studied, four genes, including *Xbp-1, sXbp-1, Chop, and Grp94*, showed a trend toward downregulation in the PFC of MS group; however, none of them were significantly downregulated. On the other hand, significant downregulation in all six UPR genes was noted in the hippocampus when the control group was compared with the MS group. Under the enriched conditions, these genes did not improve, showing their ineffectiveness in reversing the changes induced by maternal separation.

**Conclusion:**

In conclusion, our study indicates that ELS disrupts the UPR specifically in the hippocampus, suggesting a stress-induced, region-specific molecular signature linked to depression pathophysiology. On the other hand, changes in hippocampal and prefrontal UPR gene expression remain evident in the MS-enrichment group, suggesting that environmental enrichment can improve these changes but does not fully reverse them.

## Introduction

Major depressive disorder (MDD) is a prevalent and debilitating psychiatric disorder that is associated with depressed mood, apathy, executive dysfunction, and complex behavioral changes (1–3). According to the Centers for Disease Control and Prevention, depression is prevalent in 21.5% of Americans aged 18–24 years, along with a one-in-five chance that an adult will be diagnosed with depression (4). Notably, suicidal ideation is a prominent comorbid symptom of MDD, with the occurrence of a suicidal attempt at approximately 16% (5–8). Furthermore, a severe risk factor of developing MDD is early-life stress (ELS) (9–12). The stressors associated with ELS can result in profound structural and functional changes in the brain, disrupting many key emotional, cognitive, and behavioral processes. It has been suggested that ELS is detrimental to the development of neuronal plasticity, the organization of synapses, and the advancement in behavioral and psychological cognition (13, 14). Such effects can negatively shape behavioral functioning in adulthood. These neural and cognitive deficits may be further exacerbated by early-life stressors, including physical, emotional, or sexual abuse, thereby increasing the risk of psychiatric disorders such as MDD, borderline personality disorder, substance use disorder, and anxiety disorders (13, 15–18). While significant progress has been made in linking ELS to MDD, the precise molecular mechanisms remain poorly understood. ELS exerts widespread effects on the brain by altering stress-regulatory neuroendocrine systems, promoting neuroinflammation, and inducing lasting epigenetic modifications (13, 19, 20). Converging evidence suggests that large-scale gene regulatory changes underlie most, if not all, structural and functional abnormalities in the brain and are themselves targets of transcriptional dysregulation. These disruptions contribute to structural and functional reorganization within neural circuits governing anxiety and depression, particularly in regions such as the prefrontal cortex (PFC) and hippocampus (21–25).

It is increasingly recognized that brain function emerges from dynamic interactions across distinct regions. In this regard, PFC and hippocampus play central roles in the development and persistence of depression and are particularly vulnerable to the effects of early-life and chronic stress (21–25). Stress-induced disruptions in these two susceptible brain regions are associated with cognitive and emotional dysregulation, along with memory impairments (26–28). In MDD, both areas exhibit not only individual dysfunction but also show altered connectivity, reflecting their unique and shared involvement in mood regulation (29–31). Among the molecular mechanisms proposed to underlie these changes, our previous research showed that the unfolded protein response (UPR) could be a major contributor to functional disturbances in the PFC and hippocampus, particularly in the context of maladaptive stress responses (32).

UPR is an adaptive inflammatory response triggered by the accumulation of misfolded proteins in the endoplasmic reticulum (ER) (33). Events that can result in protein misfolding include, but are not limited to, ER disruption and calcium homeostasis, increased protein synthesis, hypoxia, and viral infiltration (34, 35). The high binding affinity of GRP78 to misfolded proteins elicits three branches of the UPR: activating transcription factor 6 alpha (ATF6α), inositol-requiring protein-1 alpha (IRE1α), and protein kinase RNA-like ER kinase (PERK) (36). Previously, our laboratory examined gene expression changes associated with the UPR in the hippocampus of chronically restrained-stressed rats. The study found significant changes in key genes associated with a functionally active UPR system (37). This initial study serves as a cornerstone for further exploration of how UPR shapes molecular processes linked to ELS and its long-term behavioral consequences in adulthood. A recent study demonstrated that repeated maternal separation (MS) in rats led to sustained upregulation of HSPA5 and HSPA1B, molecular chaperones of the 70-kDa heat shock protein family, in both the medial PFC and blood (38). Together, these findings suggest that ELS may influence ER stress and UPR signaling throughout development, providing a potential mechanism linking early adversity to long-term behavioral vulnerability. Despite its significance, the findings have yet to be replicated, and the specific relationship between UPR-driven inflammatory effects and MDD remains to be unraveled.

The present study utilized the ELS-induced rodent model of depression involving the separation of pups from their mothers (MS) and subjected MS rats to environmental enrichment (MS-E) to explore the expression of genes associated with the UPR (*Xbp1, sXbp1, Atf4, Atf6, Grp94*, and *Chop*) in the PFC and hippocampus. Since ELS-induced depression has not been adequately studied, establishing a correlation between ELS-associated MDD and the genes affiliated with UPR can advance our understanding of potential targets of MDD and provide future therapeutic options for treating depression. In addition, investigating whether environmental enrichment triggers a significant reversal in UPR gene expression after ELS may provide insight into the remediation of depressive behaviors.

## Materials and methods

All experiments were performed in accordance with the National Institutes of Health (NIH) guide for the care and use of laboratory animals and were approved by the University of Alabama at Birmingham’s Animal Care Committee (IACUC).

### Maternal separation

**Figure 1:**
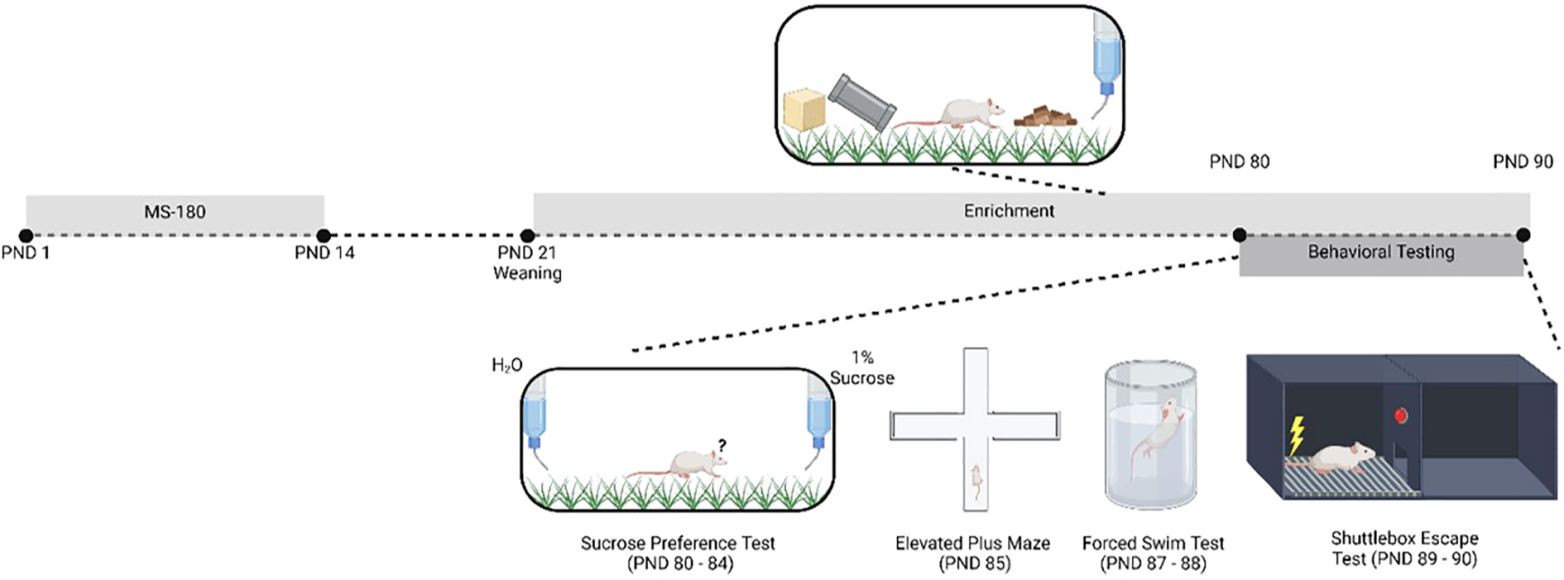
Diagram recapitulating the development of the rat ELS model. Pregnant female Holtzmann rats were housed under standard laboratory conditions for one week prior to the experiments. Rat pups were born (PND 0). Male pups were immediately distributed into control and MS groups (the MS group was further divided into EE and non-EE groups). From post-natal day (PND) 1-14, rats in the control group were handled for five minutes daily, while rats in the MS group were separated from the dams for 180 minutes and were not subject to handling. On PND 21, all rats were weaned. From PND 21-90, the MS-E rats received a form of EE. Beginning on PND 80, all rats underwent behavioral testing, including SPM, (PND 80-84), EPM (PND 85), FST (PND 87-88), and SET (PND 89-90). ELS: early-life stress; PND: post-natal day; MS: maternal separation; EE: environmental enrichment; MS-E: maternal separation with environmental enrichment; SPM: sucrose preference test; EPM: elevated plus maze; FST: forced swim test; SET: shuttle-box escape test.

Pregnant Holtzman rats were obtained from Envigo (Indianapolis, IN, USA) and were housed under standard laboratory conditions (temperature 21 ± 1°C, humidity 55 ± 5%, 12-hour light/dark cycle). Animals were given unlimited access to food and water and acclimated to the laboratory environment for one week before the experiments began. Dams were monitored twice daily from gestational day 20 until the day of birth. After the pups were born, the litters were randomly divided into two groups: the control group (n=7) and the MS group (n=14). Pups in the control group were handled for five minutes each day from postnatal day (PND) 1–14. Pups in the MS group were separated from the dam and housed individually for 180 minutes daily from PNDs 1-14. After PND 14, the pups were housed with the dam until weaning at PND 21. A schematic diagram used to develop an animal model of ELS is displayed in **Figure 1**. The study included male rats only.

### Environmental enrichment

A subset of MS animals (MS-E, n=7) was randomly assigned to receive environmental enrichment from PND 21 to 90. Environmental enrichment consisted of colored toys, tubes, shreddable objects, and manzanita wood within standard housing. The novelty of the enrichment objects was maintained through weekly rotation. The protocol was chosen on the basis of findings of previous studies that implemented similar interventions (39–42). Those animals assigned to no enrichment were housed in conventional housing.

### Behavior testing

Each rat was tested for depression- and anxiety-related behaviors using sucrose preference (SPT), elevated plus maze (EPM), and forced swim test (FST) as reported earlier (43).

#### Sucrose preference test (PND 80-84)

Rats were subjected to the SPT to assess the degree of anhedonia. Initially, the rats were introduced to two 500-mL bottles of 1% sucrose water for 24 hours, followed by one bottle of sucrose water and one bottle of tap water for each rat. Food and water were then withheld for 24 hours. Subsequently, each rat was individually housed with access to food and two 500-mL bottles, one containing tap water and the other containing sucrose water (1%, w/v). After a period of 24 hours, the volume of liquid consumption was measured. Sucrose preference was calculated as the percentage of sucrose water consumed relative to the total liquid intake, based on the following calculation:


$$\frac{mL\;of\;sucrose\;water}{(ml\;of\;sucrose\;water\;+\;mL\;of\;tap\;water}\;\times 100\%$$

#### Elevated plus maize (PND 85)

Following a 30-minute adjustment period in the testing room, each rat was individually positioned at the center of a raised plus-shaped platform (50 cm x 50 cm) with two open and two walled arms (15 cm tall, open roof). For five minutes, the animals were recorded using Noldus Ethovision XT 11.5. Open and closed arm time and frequency were extracted using the software. An anxiety index considering the frequency of entry in open arms, the frequency of total arm entry, and the duration of the test was calculated. The formula used for the calculation is as follows:

#### Forced swim test (PND 87-88)

Twenty-four hours before testing, each animal was accustomed to a Plexiglas cylinder (~ 30 cm x ~ 40 cm) filled with ~ 20 cm of room temperature water (~25°C) for 15 minutes. During the testing phase, the subjects swam under identical conditions each day for 6 minutes. The entirety of each session was video recorded and analyzed using Kinoscope software. Immobility, swimming, and climbing scores were extracted.

#### Shuttle-box escape test (PND 89)

Twenty-four hours after the rats underwent the EPM, escape latency was measured using a shuttle-box (70 cm x 20 cm x 20 cm) that was furnished with an electrified grid floor. A foot shock was generated by a shock generator and administered through the grid floor (0.6 mA on a variable interval schedule). Over five trials, the testing procedure involved a single crossing, which terminated the electrical shock. The protocol then required for the animal to cross and then to return to the initial side for the shock to cease. This test occurred over twenty-five trials. The electrical shock was applied for 30 seconds after each trial. The latency for escape was recorded.

### Tissue collection (PND 90)

Twenty-four hours after the last test, rats were anesthetized using an isoflurane vaporizer. Isoflurane was delivered through inhalation at 5% mixed into oxygen until the animal was fully unconscious. Anesthesia was maintained until euthanasia via bilateral thoracotomy. Trunk blood was collected directly from the heart in EDTA tubes. Whole blood was centrifuged (1400 rpm for 15 minutes at 4°C) to collect the plasma. The rat brain underwent regional dissection and was flash frozen in liquid nitrogen. After dissection, the PFC and hippocampi were stored at -80°C until analysis. Corticosterone was measured in rat plasma collected at the time of sacrifice using an enzyme-linked immunosorbent assay (Enzo Life Sciences).

### RNA isolation

Using the protocol outlined in our previous study (44), total RNA was isolated using TRIzol^®^ (Invitrogen Life Technologies, USA). Following phase separation with chloroform (200 μL), the aqueous phase (500 μL) was obtained. Glycogen-aided RNA precipitation used 510 μL of isopropanol and 20 µg of glycogen. The reaction proceeded overnight at -30°C. Following 15-hour incubation, the RNA pellet was observed via centrifugation (14000 rpm, 20 minutes at 4°C) and was washed twice with 70% ethanol. Post-washing, the RNA pellet was dried before resuspension in 21 μL nuclease-free water. The RNA sample ratio designating purity (260/280 nm and 260/230 nm absorbance ratios) was determined with NanoDrop (ThermoScientific, Waltham, MA). The UV spectrophotometry-based (NanoDrop) RNA quantity and purity profiles from PFC and hippocampus are presented in **Supplementary Tables S1** and **S2**. The RNA quality was further assessed via 1% agarose gel electrophoresis.

### First-strand cDNA synthesis

First-strand complementary DNA (cDNA) was synthesized using 1 µg of isolated RNA following an oligo dT-based priming method assisted by M-MLV Reverse Transcriptase (Invitrogen, NY, USA) (43). The oligo dT-primer was annealed to the RNA in the presence of deoxynucleotide triphosphates (dNTPs). The reaction mixture was incubated at 65°C for five minutes. The reaction was then terminated for two minutes at 4°C. The reverse transcription step was performed through the addition of 5X First Strand Synthesis Buffer, DTT, RNase Out, and M-MLV Reverse Transcriptase. The reaction was incubated for 50 minutes at 37°C before being terminated at 70°C for fifteen minutes.

### qPCR primer design

Primers for the coding gene were designed using rat-specific sequences sourced from the Rat Genome Database. The specificity of the primer sequences was assessed via a BLAST search against the NCBI nucleotide database and optimized accordingly to eliminate potential off-target amplification. The designed primer sequences were custom-synthesized using IDT oligo synthesizing service (IDT Technologies, NC, USA). The primers used in the expression study are detailed in **Table 1**.

**Table 1:** mRNA primers for qPCR-based gene expression analysis.

Gene	Forward (5’-3’)	Reverse (5’-3’)
*Actb*	AGG AGT ACG ATG AGT CCG GC	AAA CGC AGC TCA GTA ACA GTC
*Atf4*	CAC TGA GCA TCT CCC TCA CAA	TGG TAT TCG AGA GAA GGG AGG
*Atf6*	AGG AGT ACG ATG AGT CCG GC	AAA CGC AGC TCA GTA ACA GTC
*Xbp1*	AAA CGG CAA CTC TCC GGT CA	TTA AGC TGA GGC GGA GCA TC
*sXbp1*	AAG GCA GAT TCT CTC GCC AA	TTC TTC CCC CTT GCC TTA CG
*Chop*	CGA GGG AGA GGT GTC TGT TTC	GTC TTC ACC TGG TCC ATG AGG
*Grp94*	CCA CTT GGT ACA GAC CAC TCC	AGA CAC TAA TCA GCT GGG GG

### qPCR-based gene expression in rat PFC and hippocampus

Relative mRNA (gene) expression was examined by qPCR in combination with 1X EvaGreen qPCR master mix (Applied Biological Materials, Richmond, Canada). The genes selected (*Xbp1, sXbp1, Atf4, Atf6, Grp94, Chop*) are either involved in the direct pathway of UPR, catalyzed by UPR, or are molecular chaperones that can induce or halt the UPR response (37). Forward and reverse mRNA primers specific to rats were evaluated at a concentration of 0.5 µM.

The raw cDNA was diluted 40-fold and utilized for qPCR amplification. The procedure employed for qPCR amplification consisted of an initial denaturation stage at 95 °C for ten minutes, followed by 40 consecutive cycles of denaturation at 95 °C for ten seconds, a primer annealing stage at 60 °C for fifteen seconds, and a cDNA extension stage at 72 °C for twenty seconds. Gene expression levels were normalized to *β-actin* (*Actb*) expression values. The fold change of each selected gene was determined with Livak’s ΔΔCT method (45).

### Statistical analysis

The behavioral data were analyzed using a Student’s t-test (SPSS) to evaluate the effects of ELS and environmental enrichment on rats and to detect any significant changes that resulted from the stress paradigm. Additionally, the same test was used for data analysis to examine the expression of genes and to identify any significant variations that could indicate the expression of the UPR pathway. Data were first assessed for normality. For data that followed a normal distribution, statistical comparisons were performed. Levene’s Test for Equality of Variances was conducted using SPSS (IL, USA) to assess the homogeneity of variances and determine the degrees of freedom (*df*) among the control and experimental groups. The significance level for all analyses was set at p ≤ 0.05.

## Results

### Animal behavior

**Figure 2:**
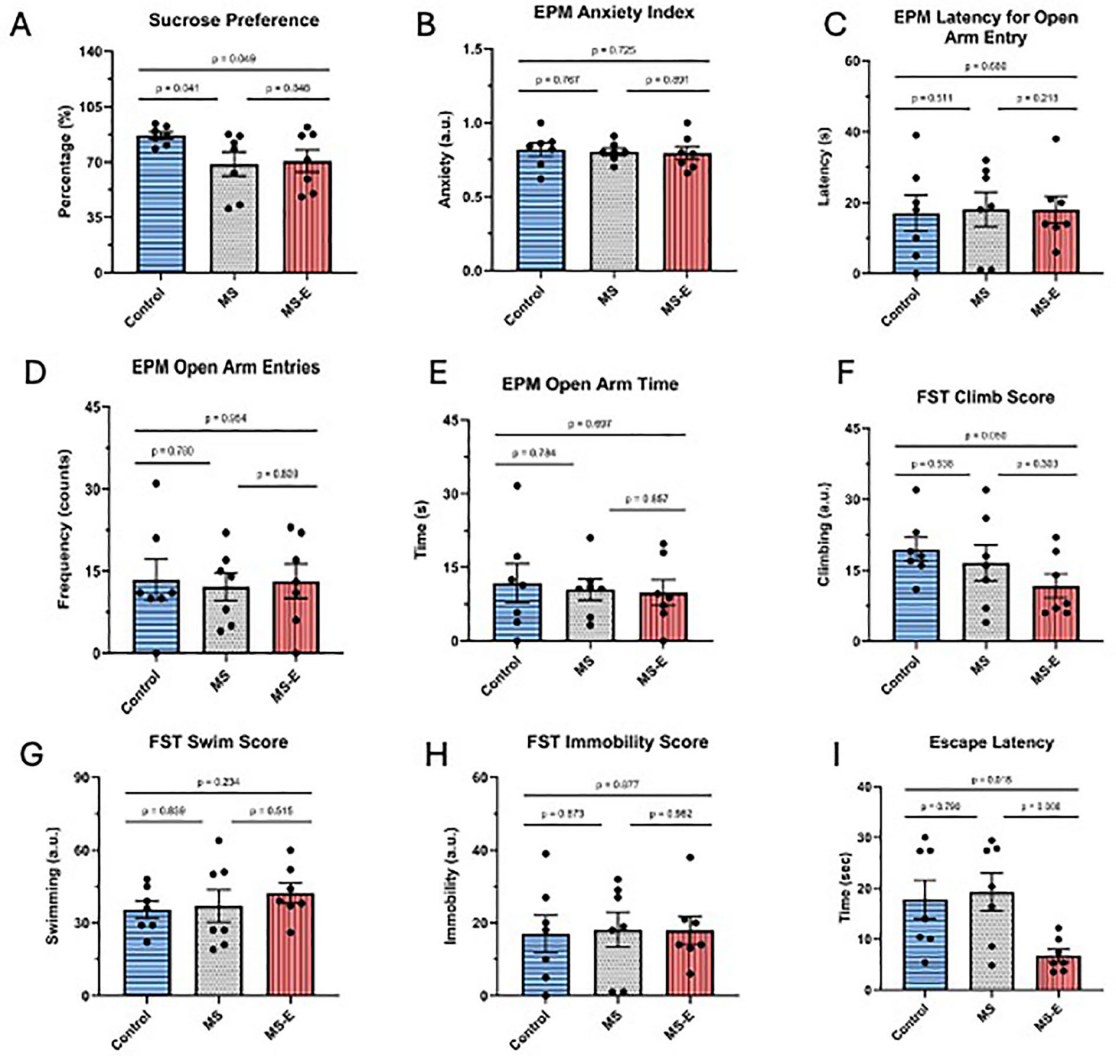
Behavioral assessment following maternal separation and environmental enrichment. The male rats subjected to maternal separation (PND1-PND14) with or without subsequent environmental enrichment were assessed for behavioral alterations at the indicated postnatal days. Behavioral tests included the sucrose preference test (SPT; PND80-84), elevated pluz maze (EPM; PND85), forced swim test (FST; PND87-88) and shuttle-box escape test (SET; PND89). **(A)** Sucrose preference test (SPT); following SPT, animals in the MS group showed significant decrease when compared to the control group. While there was no significant variation when comparing the MS group and the MS-E group, sucrose preference was significantly reduced when MS-E was compared to control. **(B)** Elevated plus maze (EPM) anxiety index; no significant change was found in MS and MS-E groups after calculating the anxiety index for EPM. **(C)** Elevated plus maze (EPM) latency to open arm; the latency to enter the open arm was not found to be significant when the control group was compared to the MS group; furthermore, despite an increase in latency, insignificant changes were found when the MS group and the MS-E group were compared. **(D, E)** Elevated plus maze (EPM) open arm entries & open arm time; no significant changes were observed between groups in the number of entries into the open arms of the EPM or the time spent in the open arms. **(F-H)** Forced swim test (FST) climb score, swim score & immobility score; In the FST, climbing score did not differ significantly between control and MS groups. Climbing scores were lower but insignificant in the MS-E group compared to the MS group. Swimming score and immobility score did not differ significantly between control and MS groups or between MS and MS-E groups. **(I)** Shuttle-box escape test (SET); escape latency in the SET did not differe significantly between control and and MS groups. Following environmental enrichment, the MS-E group showed a significant reduction in the escape latency compared with the MS group. Escape latency was also significantly lower then in the control group. MS: maternal separation; MS-E: maternal separation with environmental enrichment, MS (*n* = 7); MS-E (*n* = 7). The mean behavior differences, calculated by a Student’s t-test, are shown as dot plots. Data are mean ± SEM.

Sucrose preference was significantly lower in the MS group compared with the control (*p* = 0.041, *F* = 14.122, *df* = 12). While no significance was reported when comparing MS to MS-E, a significant downward trend was seen when comparing sucrose preference between the control group and the MS-E group (*p* = 0.049, *F* = 12.536, *df* = 12) (**Figure 2A**). The EPM anxiety index, which incorporates several EPM values into a single numerical statistic, was not significant among control and MS (*p* = 0.767, *F* = 1.429, *df* = 12), as well as MS and MS-E rats (*p* = 0.891, *F* = 1.772, *df* = 12) (**Figure 2B**). A similar nonsignificant trend was detected when the control group was compared with the MS group, and when the MS group was compared with the MS-E group in EPM latency for open arm entry (*p* = 0.511, *F* = 4.062, *df* = 12) (*p* = 0.218, *F* = 6.569, *df* = 12), EPM open arm entries (*p* = 0.780, *F* = 0.412, *df* = 12) (*p* = 0.809, *F* = 0.189, *df* = 12), EPM open arm time (*p* = 0.784, *F* = 1.551, *df* = 12) (*p* = 0.857, *F* = 0.438, *df* = 12), FST climbing (*p* = 0.538, *F* = 1.139, *df* = 12) (*p* = 0.303, *F* = 0.598, df = 12), FST swimming (*p* = 0.839, *F* = 7.777, *df* = 12) (*p* = 0.515, *F* = 4.647, *df* = 12), and FST immobility scores (*p* = 0.873, *F* = 0.013, *df* = 12) (*p* = 0.982, *F* = 0.548, *df* = 12) (**Figures 2C-H**). However, when comparing the effects of MS-E to control rats, there was a significant decrease in the FST climbing score (*p* = 0.050, *F* = 0.309 *df* = 12, **Figure 2F**). Evaluating the escape latency of the SET, no significant time difference was observed between the control group and the MS group. However, a significant reduction was found when comparing MS-E rats and MS rats (*p* = 0.008, *F* = 8.405, *df* = 12). Additionally, a decrease in escape latency was significantly expressed when comparing the control group and the MS-E group (*p* = 0.018, *F* = 24.463, *df* = 12) (**Figure 2I**). Across the behavioral tests where MS-E animals were assessed, including the elevated plus maze, open field test, FST swimming, and FST immobility, no significant differences were observed compared to controls.

### UPR-associated gene expression changes

**Figure 3:**
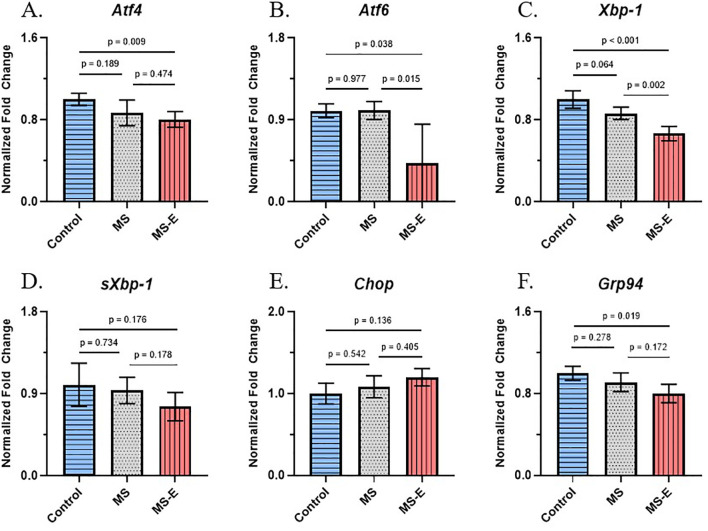
UPR-associated gene expression profile of *Atf4*, *Atf6*, *Xbp-1*, *sXbp-1*, *Chop*, and *Grp94* mRNAs in the PFC following maternal separation and environmental enrichment. Male rats subjected to maternal separation (PND1-PND14) with or without subsequent environmental enrichment were used for gene expression analysis. PFC tissue was collected at PND90. Fold change was calculated (SPSS) and is shown via the bar diagrams via Student’s t-test. Data are the mean ± SEM. **(A)** No significant change in mRNA expression was found between control and MS or MS and MS-E rats. **(B)** Significant downregulation was found after MS rats were exposed to EE (*p* = 0.015, *F* = 30.450, *t* = -2.189, *df* = 12). There was no observation of significant change between MS and control rats (*p* = 0.977, *F* = 0.438, *t* = 0.030, *df* = 10). Drastic downregulation was seen in MS rats (*p* = 0.064, *F* = 0.148, *t* = -2.059, *df* = 11). After EE, significant downregulation was observed (*p* = 0.002, *F* = 0.688, *t* = -4.076, *df* = 12). **(D-F)** No significant change was found in MS or MS-E rats for *sXbp-1*, *Chop*, or *Grp94*. Data was normalized against *Actb*. *p* ≤ 0.05. MS: maternal separation; MS-E: maternal separation with environmental enrichment; EE: environmental enrichment; control (*Atf4*, *Xbp-1*, *sXbp-1*, *Chop*, *Grp94*: *n* = 6; *Atf6*: *n* = 5); MS (*n* = 7); MS-E (*n* = 7).

**Figure 4:**
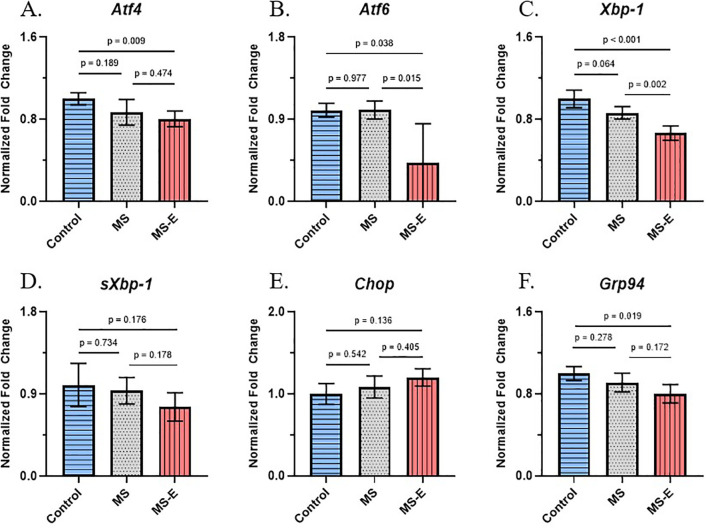
UPR-associated gene expression profile of *Atf4*, *Atf6*, *Xbp-1*, *sXbp-1*, *Chop*, and *Grp94* mRNAs in the hippocampus following maternal separation and environmental enrichment. Male rats subjected to maternal separation (PND1-PND14) with or without subsequent environmental enrichment were used for gene expression analysis. PFC tissue was collected at PND90. Fold change was calculated (SPSS) and is shown via the bar diagrams via Student’s t-test. Data are the mean ± SEM. **(A)***Atf4* showed significant expression reduction between control and MS rats (*p* = 0.017, *F* = 3.270, *t* = -2.852, *df* = 10). Between MS and MS-E rats, there was downregulation observed, but it did not reach significance (*p* = 0.091, *F* = 4.733, *t* = -1.868, *df* = 10). **(B)** Significantly reduced mRNA transcripts were found in *Atf6* in MS (*p* = 0.004, *F* = 0.446, *t* = -3.284, *df* = 10), but significance was not found when comparing MS-E to MS (*p* = 0.084, *F* = 5.956, *t* = -1.917, *df* = 10). **(C)***Xbp-1* reached significant downregulation in MS rats (*p* = 0.003, *F* = 1.039, *t* = -3.936, *df* = 10). **(D)** When comparing MS and control rats, *sXbp-1* showed significantly reduced levels of mRNA transcripts (*p* = 0.008, *F* = 2.204, *t* = -3.284, *df* = 10). **(E)***Chop* expression was significantly reduced after MS exposure (*p* = 0.008, *F* = 2.313, *t* = -3.274, *df* = 10). **(F)***Grp94* displayed a significant decrease in gene expression when comparing MS to control rats (*p* = 0.001, *F* = 0.718, *t* = -4.327, *df* = 10). After EE protocol, *Grp94* showed reduced expression within the MS-E group, but was *p* > 0.05. (*p* = 0.084, *F* = 6.787, *t* = -1.922, *df* = 10). Data was normalized against *Actb*. *p* ≤ 0.05. MS: maternal separation; MS-E: maternal separation with environmental enrichment; EE: environmental enrichment; control (*n* = 6); MS (*n* = 6); MS-E (*n* = 6).

Expression analysis of six genes (*Xbp1, sXbp1, Atf4, Atf6, Grp94, Chop*) was performed in the PFC and the hippocampus following qPCR. The normalized ΔCT values of each of the genes analyzed in the PFC and hippocampus are displayed as dot plot bar diagrams in **Supplementary Figures S1**, **S2**, respectively. The ΔCT values for each sample were averaged within the control, MS, and MS-E groups to obtain the mean ΔCT for each group. In a comparison to the control group, fold change values were determined for both the MS and the MS-E groups. Further, in a comparison to the MS group, fold change values were determined for the MS-E group. The respective fold change values for each UPR gene are illustrated via bar diagrams and collectively presented in **Figures 3**, **4**. All qPCR data for the six UPR genes (*Atf4, Atf6, Xbp-1, sXbp-1, Chop, and Grp94*), derived from group comparisons, are presented in **Supplementary Table S3**.

### UPR-associated gene expression changes in prefrontal cortex

In the PFC, the expression levels of *Atf4* were not significantly different between the control group and the MS group (*p* = 0.189, *F* = 0.803, *t* = -1.400, *df* = 11). The comparison between the MS group and the MS-E group was insignificant as well (*p* = 0.474, *F* = 0.503, *t* = -0.740, *df* = 12) (**Figure 3A**). Additionally, the fold change of *Atf6* displayed no evidence of significant change between MS and control rats (*p* = 0.977, *F* = 0.438, *t* = 0.030, *df* = 10). However, when comparing MS rats to MS-E rats, there was a significant 57.49% downregulation of *Atf6* (*p* = 0.015, *F* = 30.450, *t* = -2.189, *df* = 12) (**Figure 3B**). *Xbp-1* gene showed a trend of downregulation, albeit nonsignificant, between MS rats and control rats (*p* = 0.064, *F* = 0.148, *t* = -2.059, *df* = 11), however, it was significantly decreased by approximately 23% in the MS-E group when compared with the MS group (*p* = 0.002, *F* = 0.688, *t* = -4.076, *df* = 12; **Figure 3C**). The expression levels of *sXbp-1*, *Chop*, and *Grp94* were not significant when the MS group was compared with the control group (*sXbp-1*: *p* = 0.734, *F* = 0.518, *t* = -0.349, *df* = 11; *Chop*: *p* = 0.542, *F* = 0.561, *t* = 0.629, *df* = 11; *Grp94*: *p* = 0.278, *F* = 0.018, *t* = -1.140, *df* = 11), or when the MS group was compared with the MS-E group (*sXbp-1*: *p* = 0.178, *F* = 0.166, *t* = -1.431, *df* = 12; *Chop*: *p* = 0.405, *F* = 0.663, *t* = 0.863, *df* = 12; *Grp94*: *p* = 0.172, *F* = 0.007, *t* = -1.454, *df* = 12) (**Figures 3D-F**).

### UPR-associated gene expression changes in the hippocampus

Gene expression analysis in the hippocampus is shown in **Figure 4**. There was a significant 61.76% downregulation of *Atf4* gene between the MS group and the control group in *Atf4* (*p* = 0.017, *F* = 3.270, *t* = -2.852, *df* = 10). Although a trend of decrease in *Atf4* gene was noted in the MS rats in comparison to MS-E rats, this decrease was not significant (*p* = 0.091, *F* = 4.733, *t* = -1.868, *df* = 10, (**Figure 4A**). *Atf6* showed a comparable trend in gene expression, with significant downregulation in MS rats compared to control rats, resulting in a 56% decrease (*p* = 0.004, *F* = 0.446, *t* = -3.284, *df* = 10). No significant differences were observed in the *Atf6* gene when the MS group was compared with the MS-E group (*p* = 0.084, *F* = 5.956, *t* = -1.917, *df* = 10, **Figure 4B**). *Xbp-1* showed significant downregulation in MS rats compared with control rats (57.27% decrease, *p* = 0.003, *F* = 1.039, *t* = -3.936, *df* = 10). This gene did not show significant differences between the MS and MS-E groups (*p* = 0.118, *F* = 0.045, *t* = -1.708, *df* = 10, **Figure 4C**). In *sXbp-1*, a significant downregulation in the MS group was noted, with an approximately 50% reduction in expression (*p* = 0.008, *F* = 2.204, *t* = -3.284, *df* = 10), but again, comparison of the MS group with MS-E did not yield significant differences (*p* = 0.345, *F* = 3.868, *t* = -0.991, *df* = 10, **Figure 4D**). Another UPR gene, *Chop*, was significantly downregulated in the MS group compared to the control group (70.10% decrease, *p* = 0.008, *F* = 2.313, *t* = -3.274, *df* = 10; **Figure 4E**). *Grp94* was also significantly downregulated in the MS group when compared to the control group (61.04% decrease, *p* = 0.001, *F* = 0.718, *t* = -4.327, *df* = 10). The expression level of *Grp94* was not statistically different between MS rats and MS-E rats (*p* = 0.084, *F* = 6.787, *t* = -1.922, *df* = 10, **Figure 4F**).

### Plasma corticosterone level

There was no significant effect of MS on corticosterone levels when compared with control rats. Similarly, comparing MS+E separately with both control and MS groups did not reveal notable changes in corticosterone levels, and the differences were not statistically significant. The results are shown in **Supplementary Figure S3**.

## Discussion

This study provides the first comprehensive report on the role of the UPR system in an MS model. The findings reveal region-specific alterations in UPR gene expression. In the PFC, only Xbp-1 showed a trend toward downregulation, although it was not statistically significant, while other genes showed no changes. In contrast, the hippocampus exhibited a consistent and marked downregulation of all six UPR-related genes analyzed—*Xbp-1*, *sXbp-1*, *Atf4*, *Atf6*, *Grp94*, and *Chop* —highlighting a broader vulnerability of this region to ELS. Notably, despite the previously reported behavioral benefits of enrichment (46–49), there was no evidence of reversal in either brain region. These findings suggest that ELS leads to persistent impairments in UPR signaling, particularly in the hippocampus, which may contribute to long-term susceptibility to depression.

UPR is a cellular stress response activated in the ER to restore protein-folding homeostasis when misfolded proteins accumulate. It is comprised of three primary signaling branches, each initiated by a distinct ER-resident sensor: IRE1α, PERK, and ATF6α (36, 50). Upon activation, IRE1α mediates the splicing of *Xbp-1* mRNA to produce the active form of *Xbp-1*, sXBP-1, which enhances the expression of genes involved in protein folding and degradation (51–53). The PERK pathway functions through the phosphorylation of eukaryotic initiation factor 2 alpha (eIF2α), which reduces global protein translation while selectively increasing *Atf4* translation, a transcription factor involved in stress adaptation and apoptosis (54, 55). ATF6α, upon ER stress, translocates to the Golgi apparatus, where it is cleaved to produce a cytosolic fragment that enters the nucleus to activate UPR target genes, such as *Grp94* and *Xbp-1* (56, 57). Dysfunction in any of these branches can compromise the cell’s ability to cope with stress and has been increasingly linked to psychiatric and neurodegenerative disorders (58–61). By examining gene expression changes in the PFC and hippocampus, two key brain regions commonly implicated in depression, we provide novel insight into how ELS may disrupt cellular stress response pathways at a molecular level. Gene expression analysis revealed significant alterations in several UPR components. Within the PFC, expression of several genes showed a trend in downregulation, including *Xbp-1* and *Atf6.* In contrast, the hippocampus showed robust downregulation of all six UPR-related genes in MS rats compared with controls, suggesting a region-specific vulnerability to ELS in cellular stress regulation. One proposed mechanism for this widespread downregulation involves GRP78, or binding immunoglobin protein (BiP), an ER chaperone protein central to initiating the UPR by dissociating from ER stress sensors such as IRE1α, PERK, and ATF6α in response to misfolded proteins (36, 50). Without this dissociation, downstream transcriptional activation is hindered, potentially explaining the suppressed expression levels observed. Notably, prior work from this lab using a different depression model (restraint stress) reported upregulation of UPR genes, indicating that UPR responses may vary depending on the type and timing of stress exposure, suggesting specificity in how ELS modulates these pathways (37).

In the PFC, *Xbp-1* and *sXBP-1* did not show significant downregulation following maternal separation, although a decreasing trend was evident. This suggests a more limited or delayed UPR involvement in this region. In contrast, both transcripts were significantly reduced in the hippocampus of MS rats, with a more pronounced effect in the enrichment group, highlighting region-specific responses of UPR signaling to early-life stress (37). A similar pattern was observed with *Atf4*, a key transcription factor downstream of the PERK-eIF2α pathway (54, 55), which was significantly downregulated in the hippocampus of MS rats. Interestingly, overexpression of *Atf4* in the nucleus accumbens has been previously linked to increased depressive-like behaviors, suggesting a complex, region-specific role for this gene in mood regulation (62). However, our findings support the idea that reduced *Atf4* expression in the hippocampus may be part of a broader ELS-induced suppression of UPR activity.

Reduced activity was also seen in ATF6α, the third branch of the UPR. In MS rats, *Atf6* was significantly downregulated in the hippocampus and, after enrichment exposure, showed a strong, statistically significant reduction in the PFC. While there have not been many reports demonstrating this, one previous report suggests that *Atf6* is upregulated in depression (37) and further supports the idea that ELS produces a distinct molecular signature. The suppression of *Atf6* could interfere with downstream transcriptional responses essential for restoring ER homeostasis and might contribute to the long-term vulnerability associated with early adversity. Another important component of the UPR is its role in regulating inflammatory responses, which are closely linked to the pathophysiology of depression. In this study, *Grp94*, a major immune chaperone protein involved in folding toll-like receptors (TLRs) (63), was significantly downregulated in the hippocampus of MS rats. TLRs are known to promote pro-inflammatory cytokine release, and several (including TLR2, 4, 5, 7, and 9) require proper folding by GRP94 (63–65). The downregulation of *Grp94* may suggest a blunted or maladaptive immune response in the brain following ELS. In the PFC, *Grp94* expression was not significantly reduced in the MS group compared with controls, indicating that maternal separation alone did not affect the chaperone in this region. In contrast, a significant reduction was observed in the enrichment group, suggesting that enrichment following early life stress may differentially modulate *Grp94* expression in the PFC. Likewise, *Chop*, a pro-apoptotic factor downstream of PERK and ATF4 signaling (66), was significantly downregulated in the hippocampus of MS rats. CHOP has been associated with the production of pro-inflammatory cytokines like IL-1β and IL-23 (67, 68), suggesting that reduced *Chop* expression may reflect broader suppression of stress-induced inflammatory signaling. These findings contradict prior reports and further emphasize that UPR pathway activity in the context of ELS may differ substantially from that seen in other stress-associated depression models. Earlier studies have indicated that various stress paradigms can induce divergent gene regulatory responses that are region-specific within the brain. It has also been emphasized that the characteristics of distinct stress paradigms can induce differential regulatory responses in gene expression. Consistently, early life stress in our study elicited a divergent pattern of UPR regulation relative to our previous observations in the chronic restraint rat model.

Collectively, these results demonstrate that MS, as a model of ELS, leads to widespread downregulation of UPR-related genes in key brain regions involved in mood regulation. This pattern suggests that dysregulation of the UPR may be a key molecular mechanism underlying the long-term effects of ELS on brain function and behavior, including vulnerability to depression. While environmental enrichment has been shown to confer behavioral benefits in other contexts, our *in vivo* molecular findings indicate that it does not significantly restore UPR-related gene expression in the early-life stress model we used in this study. From our findings, we conclude that the increased gene dysregulation observed in our MS-E rat group likely reflects an enrichment-driven transcriptional adaptation mechanism rather than a direct reversal of stress-related changes. This finding also underscores the complexity of compensatory responses achieved through environmental enrichment. The compensation does not simply restore baseline molecular profiles but instead promotes more intricate gene regulatory changes in the two brain regions examined in this model (46–49). These findings underscore the need for further studies to unravel how early adversity shapes cellular stress responses and how this contributes to the development of stress-related disorders. Moreover, our present study was specifically designed and powered to examine a single well-defined maternal separation paradigm and did not directly compare it with other early-life stress models.

## Conclusion

Altogether, our research suggests an association between the genes involved in UPR and the ensuing ELS-induced depressive phenotypes. While this study highlights a strong association between UPR-related genes and ELS-induced depressive phenotypes, several limitations and future directions remain. One key limitation of this study is the exclusive use of male rats, which limits the applicability of its findings across sexes. Given the well documented role of sexual dimorphism in early life stress response, inclusion of female pups would have been beneficial. Since our previous studies have been conducted so far in male rats, we were interested to examine if similar changes occur across various stress conditions. Also, our human postmortem brain study examining UPR genes in MDD subjects did not show a sex effect (32). In the future, we plan to expand the study to include female rats to determine potential sex-specific differences in UPR gene regulation. Another limitation of our study is the absence of a control + environmental enrichment group, which restricts the interpretation of enrichment-specific effects independent of early-life stress. While our study provides valuable insight into UPR-related molecular changes, it is limited to transcript-level analyses, and corresponding protein-level analyses were not assessed. While gene expression examines alterations in signaling pathways, it is vital to determine whether these transcriptional changes correspond to modifications at the protein level. This limitation should be addressed in future studies.

## Data Availability

The original contributions presented in the study are included in the article/**Supplementary Material**. Further inquiries can be directed to the corresponding author.
