# Supplementary material for: Early life stress-induced depression reveals distinct region-specific modulation of unfolded protein response genes in the prefrontal cortex and hippocampus of rats
Source: Front Psychiatry. 2026 Mar 5;17:1747106. doi: 10.3389/fpsyt.2026.1747106 (PMC12999865; doi:10.3389/fpsyt.2026.1747106)
Supplement: Supplementary file 2 [file DataSheet1.docx]

**SUPPLEMENTARY MATERIAL**

Early-Life Stress Mediated Depressive Behavior​ Regulates the Unfolded Protein Response in the Prefrontal Cortex and Hippocampus of Male Rats

Andrew Kirk Griffin^1^, Bhaskar Roy^1^ and Yogesh Dwivedi^1^

^1^ Department of Psychiatry and Behavioral Neurobiology, University of Alabama at Birmingham, SC711 Sparks Center, 1720 2nd Avenue South, Birmingham, AL, USA


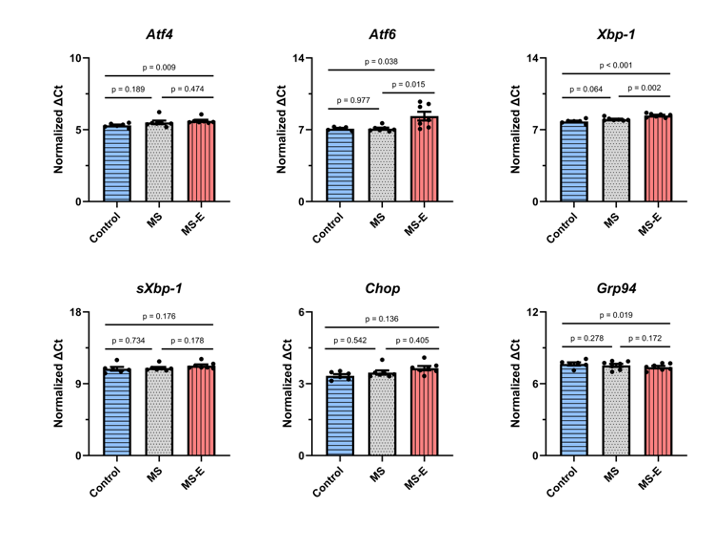
**Figure S1. UPR-associated gene expression profile of Atf4, Atf6, Xbp-1, sXbp-1, Chop, and Grp94 mRNAs in the PFC of ELS rats presented with normalized ΔCT values**. A Student’s t-test and the SPSS statistical package was utilized to calculate the ΔCT. The normalized ΔCT values for each animal are provided in the bar-dot plots. The provided data are the mean ± SEM. **A.** No significant change in mRNA expression was found between control and MS or MS and MS-E rats. **B.** Significant downregulation was found after MS rats were exposed to EE (*p* = 0.015, *F* = 30.450, *t* = -2.189, *df* = 12). There was no observation of significant change between MS and control rats (*p* = 0.977, *F* = 0.438, *t* = 0.030, *df* = 10). Drastic downregulation was seen in MS rats (*p* = 0.064, *F* = 0.148, *t* = -2.059, *df* = 11). After EE, significant downregulation was observed (*p* = 0.002, *F* = 0.688, *t* = -4.076, *df* = 12). **D-F**. No significant change was found in MS or MS-E rats for *sXbp-1*, *Chop*, or *Grp94*. This data was used to find ΔΔCT values, which was then applied to the formula 2^-ΔΔCT to determine the fold change. The data was normalized against *Actb*. *p* ≤ 0.05. MS: maternal-separation; MS: maternal separation; MS-E: maternal separation with environmental enrichment; EE: environmental enrichment; control (*Atf4*, *Xbp-1*, *sXbp-1*, *Chop*, *Grp94*: *n* = 6; *Atf6*: *n* = 5), MS (*n* = 7), and MS-E (*n* = 7).


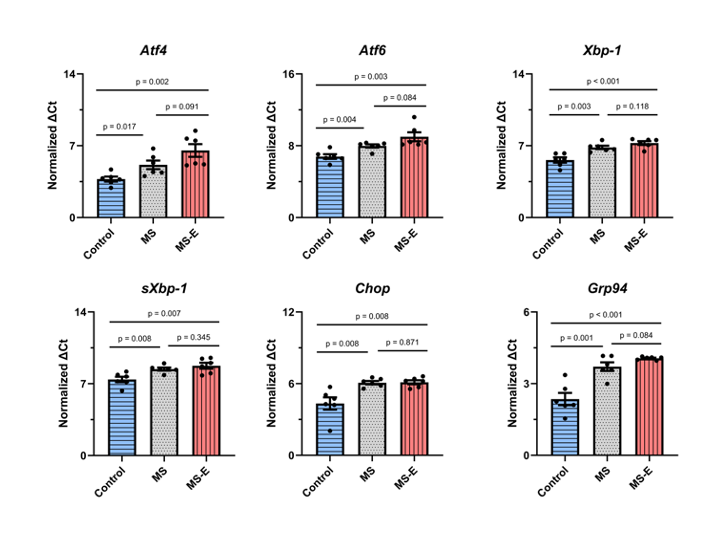
**Figure S2. UPR-associated gene expression profile of Atf4, Atf6, Xbp-1, sXbp-1, Chop, and Grp94 mRNAs in the hippocampus of ELS rats.** A Student’s t-test and the SPSS statistical package was utilized to calculate the ΔCT. The normalized ΔCT values for each animal are provided in the bar-dot plots. The data shown is the mean ± SEM. **A.** *Atf4* showed significant expression reduction between control and MS rats (*p* = 0.017, *F* = 3.270, *t* = -2.852, *df* = 10). Between MS and MS-E rats, there was downregulation observed, but it did not reach significance (*p* = 0.091, *F* = 4.733, *t* = -1.868, *df* = 10). **B.** Significantly reduced mRNA transcripts were found in *Atf6* in MS (*p* = 0.004, *F* = 0.446, *t* = -3.284, *df* = 10), but significance was not found when comparing MS-E to MS (*p* = 0.084, *F* = 5.956, *t* = -1.917, *df* = 10). **C.** *Xbp-1* reached significant downregulation in MS rats (*p* = 0.003, *F* = 1.039, *t* = -3.936, *df* = 10). **D.** When comparing MS and control rats, *sXbp-1* showed significantly reduced levels of mRNA transcripts (*p* = 0.008, *F* = 2.204, *t* = -3.284, *df* = 10). **E.** *Chop* expression was significantly reduced after MS exposure (*p* = 0.008, *F* = 2.313, *t* = -3.274, *df* = 10). **F.** *Grp94* displayed a significant decrease in gene expression when comparing MS to control rats (*p* = 0.001, *F* = 0.718, *t* = -4.327, *df* = 10). After EE protocol, *Grp94* showed reduced expression within the MS-E group, but was *p* > 0.05. (*p* = 0.084, *F* = 6.787, *t* = -1.922, *df* = 10). This data was used to find ΔΔCT values, which was then applied to the formula 2^-ΔΔCT to determine the fold change. The data was normalized against *Actb*. *p* ≤ 0.05. MS: maternal separation; MS-E: maternal separation with environmental enrichment; EE: environmental enrichment; control (n = 6); MS (n = 6); MS-E (n = 6).

**Figure S3. Plasma corticosterone levels were measured in ELS rat blood using an ELISA method.** The Differences between the groups were determined based on mean concentration, expressed in picograms per milliliter (pg/ml). The data showed a slight decrease in concentration in the MS group compared to the control group; however, this difference was not significant. In contrast, the MS+E group showed a slight increase in concentration when compared with both the control and MS groups. Likewise, no significant differences were observed. A Student’s t-test was used to determine the significance. The data shown is the mean ± SEM.

**Gene expression PCR primer profile, amplicon validation, and use of reference gene:** The PCR product sizes ranged from 75 to 150 bp, a range widely used in qPCR-based gene expression studies, particularly with dye-based amplification chemistry. All primer pairs produced single melt-curve peaks and displayed similar sigmoidal amplification plots, indicating specific amplification and comparable reaction kinetics. Collectively, these findings support the assumption that amplification efficiencies were similar across all targets. The authors selected the ΔΔCT method because it allows straightforward relative quantification of gene expression by normalizing each target gene to a reference gene, and it is widely accepted for evaluating relative gene expression changes across samples. Primer design and validation were done prior to all experimental analyses. Primers were generated using Primer3, primer design software with parameters adjusted to achieve comparable melting temperature, appropriate GC content, and minimal predicted secondary structure. The target specificity of each pair was evaluated by in silico homology search against the rat reference sequence database (NCBI). Each primer pair was then empirically validated in control samples and only primers producing a single robust amplicon under the selected cycling conditions were used in subsequent experimental analysis.

| **Table S1: Quantification of rat PFC RNA samples using Nanodrop Spectrophotometer** | | | | | | | | |
| --- | --- | --- | --- | --- | --- | --- | --- | --- |
| **Group** | **Sample ID** | **Nucleic Acid** | **Unit** | **A260 (Abs)** | **A280 (Abs)** | **260/280** | **260/230** | **Sample Type** |
| Control | Control1 | 487.8 | ng/µl | 12.196 | 6.647 | 1.83 | 2.27 | RNA |
|  | Control2 | 461.4 | ng/µl | 11.535 | 6.117 | 1.89 | 2.24 | RNA |
|  | Control3 | 463.4 | ng/µl | 11.586 | 6.376 | 1.82 | 2.12 | RNA |
|  | Control4 | 542.9 | ng/µl | 13.574 | 7.245 | 1.87 | 2.29 | RNA |
|  | Control5 | 497.9 | ng/µl | 12.447 | 6.695 | 1.86 | 1.99 | RNA |
|  | Control6 | 475.7 | ng/µl | 11.893 | 6.547 | 1.82 | 2.21 | RNA |
|  | Control7 | 510.4 | ng/µl | 12.759 | 7.052 | 1.81 | 2.22 | RNA |
| Maternal Separation | MS1 | 509 | ng/µl | 12.724 | 6.924 | 1.84 | 2.27 | RNA |
|  | MS2 | 491.4 | ng/µl | 12.285 | 6.789 | 1.81 | 2.18 | RNA |
|  | MS3 | 487.1 | ng/µl | 12.177 | 6.602 | 1.84 | 2.3 | RNA |
|  | MS4 | 560.5 | ng/µl | 14.014 | 7.635 | 1.84 | 2.29 | RNA |
|  | MS5 | 483.2 | ng/µl | 12.081 | 6.541 | 1.85 | 2.22 | RNA |
|  | MS6 | 481.6 | ng/µl | 12.039 | 6.504 | 1.85 | 2.27 | RNA |
|  | MS7 | 481.2 | ng/µl | 12.03 | 6.519 | 1.85 | 2.2 | RNA |
| Maternal Separation + Enrichment | MS+E1 | 477 | ng/µl | 11.925 | 6.423 | 1.86 | 2.07 | RNA |
|  | MS+E2 | 500.5 | ng/µl | 12.513 | 6.861 | 1.82 | 2.13 | RNA |
|  | MS+E3 | 491.7 | ng/µl | 12.292 | 6.674 | 1.84 | 2.2 | RNA |
|  | MS+E4 | 499.8 | ng/µl | 12.495 | 6.828 | 1.83 | 2.15 | RNA |
|  | MS+E5 | 494.1 | ng/µl | 12.352 | 6.687 | 1.85 | 2.17 | RNA |
|  | MS+E6 | 499.4 | ng/µl | 12.484 | 6.777 | 1.84 | 2.3 | RNA |
|  | MS+E7 | 498.9 | ng/µl | 12.472 | 6.616 | 1.88 | 1.84 | RNA |

| **Table S2: Quantification of rat hippocampus RNA samples using Nanodrop Spectrophotometer** | | | | | | | | |
| --- | --- | --- | --- | --- | --- | --- | --- | --- |
| **Group** | **Sample ID** | **Nucleic Acid** | **Unit** | **A260 (Abs)** | **A280 (Abs)** | **260/280** | **260/230** | **Sample Type** |
| Control | Control1 | 483 | ng/µl | 12.074 | 6.565 | 1.84 | 2.07 | RNA |
|  | Control2 | 469 | ng/µl | 11.725 | 6.504 | 1.8 | 2.07 | RNA |
|  | Control3 | 439.3 | ng/µl | 10.983 | 6.174 | 1.78 | 2.18 | RNA |
|  | Control4 | 418.2 | ng/µl | 10.456 | 5.56 | 1.88 | 1.3 | RNA |
|  | Control5 | 494.9 | ng/µl | 12.373 | 6.727 | 1.84 | 2.1 | RNA |
|  | Control6 | 447.2 | ng/µl | 11.179 | 6.248 | 1.79 | 2.13 | RNA |
| Maternal Separation | MS1 | 476.6 | ng/µl | 11.915 | 6.574 | 1.81 | 2.11 | RNA |
|  | MS2 | 478.3 | ng/µl | 11.958 | 6.535 | 1.83 | 2.14 | RNA |
|  | MS3 | 309.8 | ng/µl | 7.746 | 4.36 | 1.78 | 2 | RNA |
|  | MS4 | 441.9 | ng/µl | 11.048 | 5.853 | 1.89 | 1.34 | RNA |
|  | MS5 | 430.8 | ng/µl | 10.769 | 5.738 | 1.88 | 1.21 | RNA |
|  | MS6 | 457.9 | ng/µl | 11.447 | 6.295 | 1.82 | 2.11 | RNA |
| Maternal Separation+Enrichment | MS+E1 | 458.5 | ng/µl | 11.462 | 6.39 | 1.79 | 2.23 | RNA |
|  | MS+E2 | 453.8 | ng/µl | 11.344 | 6.3 | 1.8 | 2.14 | RNA |
|  | MS+E3 | 413.6 | ng/µl | 10.339 | 5.534 | 1.87 | 1.33 | RNA |
|  | MS+E4 | 420.6 | ng/µl | 10.515 | 5.559 | 1.89 | 1.23 | RNA |
|  | MS+E5 | 485.6 | ng/µl | 12.14 | 6.733 | 1.8 | 2.09 | RNA |
|  | MS+E6 | 460.6 | ng/µl | 11.516 | 6.348 | 1.81 | 1.98 | RNA |
